# A key system response of diametric space as a paradigm for understanding the phenomenology of anorexia nervosa

**DOI:** 10.3389/fpsyt.2025.1623166

**Published:** 2025-09-16

**Authors:** Paul Downes

**Affiliations:** School of Human Development, Dublin City University, Dublin, Ireland

**Keywords:** anorexia nervosa, phenomenology, diametric space, concentric space, control, perfectionism, overexercise

## Abstract

There is increasing recognition of phenomenological approaches to understanding anorexia nervosa (AN), interrogating lived experiences and voices of those diagnosed with AN. This article builds on phenomenological accounts of AN, taking a step further for a *spatial* phenomenology, to examine how specific central features of AN experience are structured in diametric spatial terms. Building on growing research on the phenomenology of AN, it is remarkable how consistently the phenomenology of AN clusters around structural features of diametric space, as reactive, compensatory coping mechanisms of splitting, closure, and mirror image inversions. Pervasive features of AN experience and motivation, including control, perfectionism, ambivalence, an anorexic “voice,” the gaze of the other, and self-hate around the body, are all aspects that cluster around the specific spatial system and process of diametric space. Diametric spatial closure from background stimuli offers a spatial concomitant of control. Restriction of food in AN may be part of a wider process of spatial constriction as the operation of a diametric spatial system of experience. This diametric spatial system of experience is in tension with a concentric spatial system, amplifying Ricoeur’s phenomenology of distantiation and Lévi-Strauss’ anthropological insights regarding the interplay between these cross-cultural structures. This conceptual, desk-based review presents a theoretical framework of AN phenomenology as displacement within experiential space, from concentric to diametric space, proposed as a prior system paving the way for bodily tensions in AN, bringing loss of capacity for concentric structured spatial experiences. This treats AN recovery as capacity for restructuring from diametric spatial oppositions to generate connective, relatively open, concentric spaces in life experiences. Developing a spatial interpretative methodology, phenomenological and qualitative AN research sources for application of this spatial analysis were selected based on their direct relevance to key aspects of DSM-5 diagnostic features for AN regarding drive for thinness, body dissatisfaction, and overexercise, as well as for central psychological understandings of AN in terms of control, identity, and perfectionism.

## 1 Introduction

Understanding anorexia nervosa as a problem of space, of being-in space, of taking up space, requires a stronger focus on modes of space in experience. In doing so, a framework is needed to concretize contrasting modes of being in space and to relate these spaces to phenomenological accounts of anorexia nervosa (AN).

The specific hypothesis being examined in this article is that in AN, diverse phenomenological features cluster into basic spatial system responses that constellate experience into diametric spatial modes of being. Diametric space involves fixed oppositions, hardened, closed boundaries, and processes of separation and splitting. A diametric spatial process and structure is interrogated as serving to restrict experience and not only restrict eating. This diametric spatial closure is proposed as shaping wholescale terrains of experience, and not only experience of the body, of exclusion from being-in the body.

This concern with modes of being for AN is resonant with a recent phenomenological metasynthesis of AN as a “state of being” [(1), p. 5]. Understanding diverse aspects of the phenomenology of AN as this recurring diametric spatial system response invites a further focus on AN recovery in terms of a shift from these diametric spatial system habits of defensive experience toward contrasting concentric spaces of experience. Diametric spatial compressions are being hypothesized as potentially malleable generative and maintaining conditions for AN, needing to be undone as part of a fluidation of experience through the softer, more connective, permeable boundaries of concentric space.

This proposed paradigm of a spatial turn for AN draws centrally on two conceptual strands for understanding fundamental spatial interactions in experience, namely, Ricoeur’s (2) and Heidegger’s (3) phenomenology and a specific aspect of cross-culturally observed spatial structures in Lévi-Strauss’ (4) structural anthropology. While previous approaches to understanding AN have tended to draw on Merleau-Ponty’s (5) phenomenology of perception and the body (6) and anthropological sources on symbolism (7) and on embodiment (8), the current framework offers a different lens.

A diverse range of AN phenomenological features pertaining to body dissatisfaction, drive for thinness, overexercise, control, identity, and perfectionism is being interrogated as clustering around diametric spatial forms, as a common spatial movement in experience. Diametric space is proposed as a self-propagating system process, as a spatial movement of its own, colonizing experience in AN; it is the spatial movement requiring direct modification.

The background diametric spatial process of constriction and disconnection provides the basis for the foreground restriction of food in AN. Anorexic restriction of food is part of a broader system of restriction of experiential stimuli through the closure of diametric space. AN recovery is proposed as part of a process of expansion of capacity for more openness to stimuli through the move toward a more connective concentric spatial process of relative opening. This invites a clinical focus for AN on developing wider spatial capacities for more open, connective spatial modes of experience, as a holistic multidimensional focus on growth in experience that is not simply on food and body-related constrictions. Experience as a spatial system is examined as a shutdown into diametric space in AN and not only regarding food and body. Within this holistic focus, wider concentric spatial system capacities for experience need to be fostered.

This framework adds a wider concern with agency at the level of shift within structured spaces of being, of being-in, in the movement from diametric to concentric spatial systems of experience. These prereflective spaces of experience share a concern with somatic approaches to AN and are wider than simply cognitive agency, though allowing also for this dimension in a more focused spatial way on axes of openness/closure, rigid mirror image oppositions in cognitions to move beyond good/bad, perfect/worthless to less sweeping cognitions that embrace relational and contextual complexity.

## 2 Diverse features of the phenomenology of anorexia nervosa: a brief overview

There is increasing recognition of a range of phenomenological dimensions to understanding AN, to interrogate the lived experiences and voices of those who are diagnosed with AN (1). These diverse phenomenological aspects cover terrains such as power and control in personal identity, tensions between subjective and objective vantage points in experience, social identity issues of success and failure, emotions of ambivalence and feeling displaced from a sense of being at home, preverbal breathing-related tensions, and existential search for meaning.

Such phenomenological research has pointed out that AN gives expression to a radical kind of Cartesian split between mind and body (7), as the individual seeks to gain control over their bodily impulses, including hunger (9). There has been a recent phenomenological focus on eating disorders as an issue of embodiment, to interrogate lived experiences of the body, contrasted as a subjective feeling state and objective external apprehension, “the basic polarity of embodiment, namely between the subjective and objective body, between one’s pre-reflective and reflective relationship to the body” [ (10), p. 110]. A related focus here is on subjective experiences of the body, where the body itself is experienced as an object (11).

Many phenomenological accounts recognize that the individual is torn between an “anorexic voice” and their own voice (12), while highlighting the issue of perfectionism associated with eating disorders, where the individual treats loss of weight in terms of social success (1). Other understandings of eating disorders explore issues of avoidance of emotion, as a strategy of repression of painful memories, as well as of ambivalence (13). This ambivalence includes a love-hate relationship with the eating disorder, as it offers a path for meaning in life, while also individuals recognize it is destroying their life. It implicates a movement between contrary states of being. Body dissatisfaction is treated in phenomenological research on AN as a multidimensional construct, including a perceived failure in personal identity and preverbal, complex emotions being “condensed down” into a feeling of being fat [ (14), p. 60]. Phenomenological research on AN, with a focus on lived experience of the body, recognizes the need to open up “substantially restricted breathing” [ (15), p. 6] in a tense body and the loss of a tacit background connection to the body that brings a separation in experience of the body, a distancing process as a detached observer, of heightened self-consciousness of the body “as if it were a separate object” [ (6), p. 234]. AN as a profound disturbance of embodiment, disproportionately experiencing one’s body as an object, subject to the evaluation gaze of the other, has been proposed as the core feature of eating disorders (16).

Themes of social and personal identity are central to eating disorders. Family systems dynamics (17) and attachment theory (18) are also viewed as key to issues of power and control, as well as to the search for connection and disconnection in AN. In addition to understandings of AN in terms of personal and social identity, another strand of research has focused on existential dimensions, where eating disorders are treated as a search for meaning in life. This includes a view of it as a generalized state of anxiety that is concretized into a focus on dissatisfaction with the body (19). The more nebulous experience of anxiety is transported into fear of a concrete object, namely, the body. Again, the theme of objectification arises. The body is experienced as a state of uncanniness, a lack of belonging, of not being at home (20).

Many of these phenomenological concerns in AN point to the need for a more fundamental interrogation of the capacity for relations *between* subjective and objective experiences of self, body, and others. Mediating spaces of betweenness need to come to the fore. This article seeks to build on phenomenological accounts of AN and to take a step further to examine them in terms of a *spatial* phenomenology (21). Whereas Descartes (22) treated space as an empty, non-entity and in terms of dualistic splits between mind and body, reason and emotion, a spatial–phenomenological approach seeks to examine space as a system, as an active relational space that need not be confined to Cartesian spatial understandings (23).

This spatial–phenomenological approach proposes an additional level of interpretation of experience, to uncover spatial patterns and regularities pervading the first-person description of experience of being anorexic. Background spatial systems of relation and meaning are construed as mediating the mind–body relation central to eating disorders. Space is treated here as being far from being simply a metaphor (21) but rather as a malleable system condition shaping modes of experience, including emotions, cognitions, and social relationships.

## 3 Rethinking the phenomenology of anorexia nervosa as a key diametric spatial system: theoretical background

### 3.1 Beyond the spatial turn for the social sciences and humanities: building on Ricoeur and the early Heidegger’s space as a fundamental phenomenological mode of being-in versus side-by-sideness

A central tenet of the spatial turn in the humanities and social sciences over the past decades has been that space is socially constructed. This initial spatial turn draws centrally on the work of Lefebvre (24) and Soja (25), treating space as conceived, perceived, and social, with Soja treating space as one that offers infinitely malleable imagined possibilities. This spatial turn highlights the blind spot of space in systems, to treat space as being far from an unmovable system feature of experience in order to reconstruct “seeming immutable forms” of space [ (26), p. 7]. There are diverse aspects within this initial spatial turn, many focusing on social spatial issues of connection and separation (27), going beyond simply treating space as physical places (28). In the words of Ferrare and Apple on this initial spatial turn, for understandings of “spatial processes … we not only need these ‘new’ theories, but we also need to employ methodological tools that ‘think’ spatially” [ (29), p. 216].

More recently, a different strand of the spatial turn, while sharing with Soja the need to challenge homogeneous space assumptions, has tended to focus on phenomenological dimensions (30), where space is more fundamental than mere social construction, than as a mere by-product of social forces. This strand of the spatial turn, contributed to by Ricoeur, builds on his readings of the early Heidegger and interrogates a background spatial realm for experience that mediates the subject–object distinction. In *Hermeneutics and the Human Sciences*, Ricoeur excavates what is tantamount to a spatial domain prior to language, while referring to an implicitly spatial preunderstanding of “distantiation” [ (2), p. 110, p. 51] as establishing the relation between subject and object. This implicates a spatial mode and generative process for the subject–object relation. Ricoeur explicitly appeals to Heidegger’s being-in-the-world as the basis for a contrast to distantiation—a contrast which Ricoeur terms “belonging” [ (2), p. 110, p. 51].

Ricoeur’s prereflective unmediated concerns with belonging are resonant with AN phenomenology concerns of Stanghellini and Mancini with “an unmediated and pre-reflexive experience of my body” [ (31), p. 146], “the primitive experience of oneself as a spatiotemporal embodied agent in the world” [ (32), p. 70] to contrast with the body perceived as object. They apprehend that in AN “what seems to be impaired is the coenaesthetic apprehension of their own body as a more primitive and basic form of self-awareness” [ (31), p. 146]. The unmediated visceral body (33) needs a mediating system space for an interplay to be-with (34) with the body perceived as object.

Ricoeur relates these spatial qualities of distance and relational connection to Heidegger’s (3) concerns as a background realm of being, of being-in-the-world, prior to subjectivity and objectivity. While Heidegger’s term being-in-the-world has been invoked in phenomenological accounts of AN (8), including with a Sartrean hue (35), for Ricoeur, “Belonging is expressed by Heidegger in the language of being-in-the-world. The two notions are equivalent” [(2), p. 106]. Ricoeur treats belonging as an immediate or unreflective relation “correcting” distantiation [ (2), p. 110, p. 51]. This is resonant with a conception of preintentional, existential feeling in psychiatry, drawing also on Heidegger’s being-in-the-world (36).

In a later work, Ricoeur explores “variations in the active and passive modes of the interplay of distantiation and closeness that makes proximity a dynamic relationship ceaselessly in motion: drawing near, feeling close”; he seeks “a range of varying distances in the relations between self and other” [ (37), p. 131]. Ricoeur examines fundamental mediating spaces between the experience of self, body, and other in subjective and objective terms. Though Ricoeur’s work is explicitly described as leading a spatial turn across many other thematic areas (38), his scrutiny of background relational space mediating experience of subjectivity and objectivity is not reducible simply to the space of embodiment. A prior spatial realm to the body is being invoked, as a background system, as a precondition for the experience of the body.

Research on AN phenomenology has already pinpointed this background relation of being, as a domain of relevance, though not doing so in directly spatial terms. Thus, Leder (34) refers to being-with as a mode between subjective and objective experience of the body. He highlights this intermediary realm for AN experience, where “the renegade body is not exactly the body-as-object nor the body-as-subject, but a kind of blended, but internally unstable, ‘object/subject’” [ (34), p. 59]. Likewise, it is recognized that there is no clear-cut subjective–objective distinction in the experience of the body as the gaze of the other affects the subjective experience of one’s body (32, 39). Again, a mediating realm between subjective and objective bodily experience is invoked. The further step being taken here phenomenologically for AN is to treat this mediating background as not only spatial but involving concrete interactive spatial systems, of diametric and concentric spaces (and monistic spatial fusion).

This concern of Ricoeur with a spatial interplay between modes of proximity, connective belonging on the one hand, and modes of distantiation, on the other hand, as ways of being-in-the-world, was anticipated in psychiatry in 1982 by Laing’s (40) phenomenological concerns with a third-order background relational truth, mediating subjectivity and objectivity. These phenomenological concerns seek a third order of patterned relation between the subjective and objective (40). The step Ricoeur adds to Laing here is that this third-order background relation to bridge the subjective and objective requires construal in terms of dynamic, contrasting relational spaces.

Ricoeur’s explicit invoking of this dynamic spatial background interplay in terms of Heidegger’s being-in-the-world invites further focus on the early Heidegger’s spatial understanding of conditions for objectivity and for being-in. For Heidegger’s (41) phenomenology, the realm of objectness is a standing opposite, a standing against, as in the German word for object, *Gegenstand*, “but instead the being *as standing-opposite*, *as standing-over-against*.” Heidegger uncovers a spatial background of opposition, a mode with “an object … counterposed to the subject” [ (41), p. 157]. This spatial counterposing in Heidegger expresses Ricoeur’s “distantiation.” Moreover, it is resonant with phenomenological concerns for AN regarding the reification process of the gaze of the other, the Sartrean shock of meeting the other (35), where the person with AN feels their body and self is objectified through the gaze of an impersonal other (31, 39). They are in a mode of standing opposite their own body, subjectively perceived as being a counterposed entity in reified terms.

**Figure 1:**
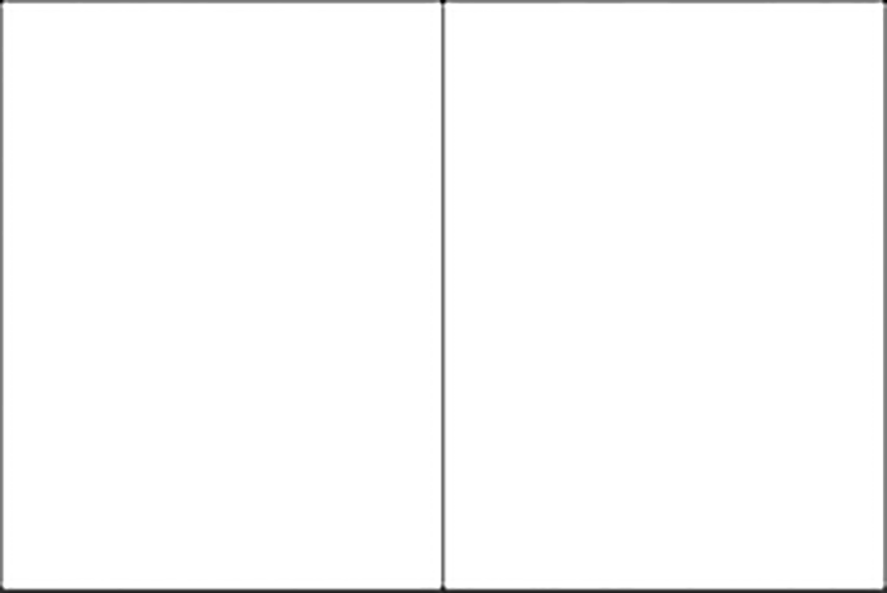
Diametric dualism.

A further step is needed here of interpreting this spatially imbued standing, this oppositional standing against, this counterpositioning, as being a concrete spatial structure of *diametric* opposition, of being a *diametric oppositional* sp*ace* of relation. In the words of Bachelard, “simple geometrical opposition becomes tinged with aggressivity” [ (42), p. 212]. This is not only a phenomenologically meaningful space; it is also to be emphasized that diametric spatial opposition is a geometrically structured space, as a diameter cutting a circle. A diametric spatial structure is one where a circle is split in half by a line, which is its diameter or where a square or rectangle is similarly divided into two equal halves (see **Figure 1**).

Understood as a geometric space, pertinent also to phenomenology, a diametric spatial opposition assumes separation between both its poles. Heidegger seeks a different space from this, a being-in that is to be fundamentally contrasted with this diametric oppositional space, “There is no such thing as the ‘side-by-side-ness’ of an entity called ‘Dasein’ with another entity called ‘world’” [ (3), p. 81]. Diametric space is where both parts are side-by-side.

Whereas Bordo (7) invokes Foucault’s (43) work to accentuate his concerns with an agonistic relation to self as part of a mastery and combat of desire, a more spatial focus in Foucault’s (44) work operates very much with an influence of Heidegger, as with Ricoeur. While there is a tendency to construe Foucault’s conceptions of space as part of a social constructionist view of space, as Soja (25) does expressly for works of Foucault, this is not the only reading of space in Foucault. Foucault’s (44) influence on the spatial turn is manifested in his concept of heterotopia, of spaces of otherness. For Foucault’s spatial turn, “The present epoch will perhaps be above all the epoch of space … we are in the epoch of juxtaposition … of the side-by-side … juxtaposed, set off against each other” [ (44), p. 22]. The underpinnings of this concept include the spatial mode of side-by-sideness that is implicitly derived from Heidegger (3). In other words, diametric oppositional space is a fundamental mode of juxtaposed space, of space being in a mode of side-by-sideness. Yet unlike Foucault, both Ricoeur and Heidegger seek a further fundamental space, a being-in, what Heidegger describes as a dwelling alongside, a more connective space of what Ricoeur terms belonging.

**Figure 2:**
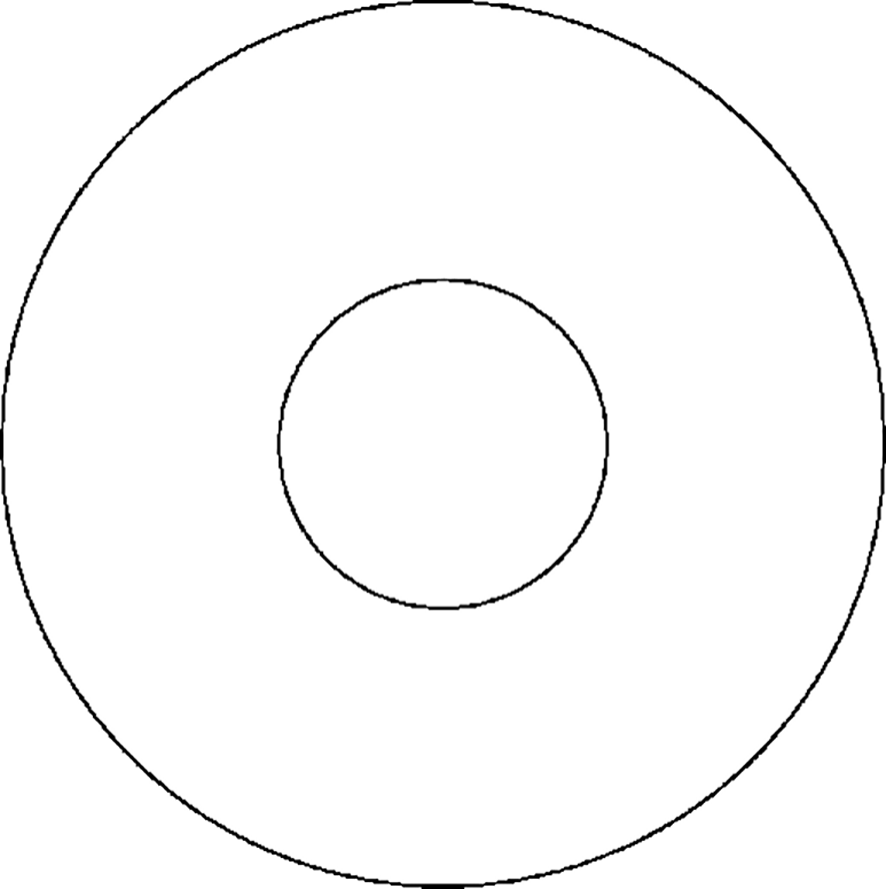
Concentric dualism.

Heidegger (3) explicitly equates the “touch” of encountering with the mode of being-in, being-alongside, dwelling alongside the world. This contrasting space to diametric opposition is to be construed as a geometrical one of concentric space. In a concentric spatial structure, one circle is inscribed in another larger circle (or square); in pure form, the circles share a common central point (see **Figure 2**), a co-center.

It is geometrically evident that the inner and outer poles of concentric structures are more fundamentally attached than diametric structures. Both concentric poles coexist in the same space around a common center so that the outer circle overlaps the space of the inner one. The outer circle surrounds and contains the inner circle. The opposite that is within the outer circle or shape cannot detach itself from being within this outer shape. Although the outer circle or shape can move in the direction of greater detachment from the inner circle, it cannot fully detach itself from the inner circle (even if the inner circle becomes an increasingly smaller proportion of the outer). A concentric relation assumes a connection between its parts, and any separation is on the basis of an assumed connection, whereas diametric opposition assumes separation and any connection between the parts is on the basis of this assumed separation (21). Concentric space offers a relation that allows for distinction between an inner and outer pole while retaining an underlying connection. The juxtaposition of diametric oppositional space is a less proximate relation between both poles than in concentric spatial relation.

Diametric space is a framing condition for Ricoeur’s distantiation, with concentric space offering a framing condition for Ricoeur’s belonging. It is notable that Ricoeur treats these spatial modes as interactive, as belonging is envisaged as correcting distantiation. This hypothesizes that an increase in one brings a corresponding decrease in the other in a compensatory fashion. Ricoeur seeks a dynamic interplay to this spatial mediating background, so that diametric and concentric spaces are processes as well as structures of experience. This implicates a capacity for a flexible, fluid common background space mediating the experience of the self as subject and object.

It is against this backdrop of a different spatial turn focusing on mediating spaces of connection and separation, as spatial ways of being-in-the-world, that the question arises as to the relevance of such phenomenological spaces and spatial concerns of belonging and distantiation to AN. This background space of being, mediating the experience of being both a subject and object of experience, requires further amplification for AN. In doing so, these spatial modes of being are to be treated as prior simply to Merleau Ponty’s (5) phenomenology of perception of the body, already examined in phenomenological research on AN (6). Space offers ways of being and not simply aspects of perception for this different phenomenological strand of the spatial turn. Similarly, this spatial focus does not reject embodiment and cultural dimensions to AN; rather, it focuses on spatial preconditions for embodiment, for being-in the body. Yet, before examining diametric and concentric spatial projections in the phenomenology of AN, two further contrasts between these spaces must be highlighted, drawing on structural anthropology.

### 3.2 Concretizing a spatial phenomenology as interactive systems between contrasting diametric and concentric spaces

Diametric and concentric spaces are not only contrasting geometric spaces in terms of assumptions of separation and connection; they have been observed as empirical phenomena in physical and social structures, as well as myths, cross-culturally by a range of anthropologists. Diametric spatial opposition has been observed cross-culturally, by Lévi-Strauss, noting that examples of diametric dualism “abound” [ (45), p. 135], citing specific tribes in North and South America. Moreover, the simple “subjective” [ (46), p. 111] everyday cross-cultural oppositions between “good” and “bad” are structured in a diametric oppositional way, as are success/failure and love/hate.

Lévi-Strauss (47) cites a range of cross-cultural observations of concentric structures of a number of anthropologists. These include the following: the village plan of Omarakana in the Trobriand Islands, published by Malinowski; the Baduj of western Java and the Negri-Sembilan of the Malay peninsula, observed by de Jong; the village of the Winnebago tribe observed by Radin; and an archeological finding in the Lower Mississippi Valley. These are ancient spatial structures. For example, with regard to the Lower Mississippi Valley, Lévi-Strauss states:

We are therefore dealing with a type of [concentric] structure which in America extends far back into antiquity, and whose later analogues were to be found in preConquest Peru and Bolivia and … in the social structure of the Sioux in North America and of the Ge and related tribes in South America … This latter structure however was not simply bipolar but forming six concentric octagonal figures. [ (47), p. 143].

A further example of the ancient features of concentric and diametric space is 3,000-year-old ring-graves (*kivikirstkalmed*) in Muuksi, Vohma, and Kasekula, Estonia, which have a concentric structure (48). This concentric structure contrasts with the later (*tarandkalmed*) graves from the third to fifth century in Jaagupi, Estonia, which have a diametric structure; from the 12th century at Kaku, in Saaremaa, Estonia, the circular, concentric structure of the burials vanishes (48).

Resonant with Ricoeur’s (2) subsequent account of a mutual tension between background spaces of distantiation and belonging, where belonging “corrects” distantiation, in a kind of compensatory relation, a key insight of Lévi-Strauss is that diametric and concentric spaces are systems in “functional relation” [ (4), p. 73]; they exist in interactive tension, where an increase in one brings a decrease in the other. Lévi-Strauss identified two key contrasts empirically between these spaces. Firstly, diametric oppositional space is a more closed one in relation to a background environment. “In a diametric system … virgin land constitutes an irrelevant element; the moieties are defined by their opposition to each other, and the apparent symmetry of their closed structure creates the illusion of a closed system” [ (47), p. 152]. This more closed system of diametric space is less permeable with more rigid boundaries that exclude interaction with the background. In contrast, Lévi-Strauss rejects closure for concentric structures, treating them as a relatively more open, interactive system with the background. Lévi-Strauss observes:

[In concentric relation] the system is not self-sufficient, and its frame of reference is always the environment. The opposition between cleared ground (central circle) and waste land (peripheral circle) demands a third element, brush or forest – that is, virgin land – which circumscribes the binary whole while at the same time extending it, since cleared land is to waste land as waste land is to virgin land. [ (47), p. 152].

Lévi-Strauss (47) highlighted that concentric spatial systems are more fluid and open, interacting dynamically with the background.

The second contrast identified by Lévi-Strauss (4) is where he explicitly relates diametric structures to mirror image inversions between both diametric poles. He describes “symmetrical inversions” [ (4), p. 247] in Mandan and Hidatsa myths:

these myths are diametrically opposed…. In the Mandan version … two earth women who are not sisters go to heaven to become sisters-in-law by marrying celestial brothers. One who belongs to the Mandan tribe, separates from an ogre, Sun, with the help of a string which enables her to come back down to her village. In revenge, Sun places his legitimate son at the head of the enemies of the Mandan, upon whom he declares war. In the Hidatsa version … everything is exactly reversed. Two celestial brothers come down to earth to be conceived by human beings and born as children. Sun’s sister, an ogress, is joined with an earthborn character by means of a string. She makes him her adopted son and puts him at the head of the enemies of the Hidatsa. [ (4), p. 250].

As Lévi-Strauss (4) documented, diametric space operates through a mirror image symmetry, an inversion between poles in stark opposition, such as good/bad, inside/outside, sacred/profane, and above/below. A mirror image is not identical but rather a left–right inversion. Concentric structures of relation are not a symmetry as inversion. Rather, they offer a different symmetry as unity, where the point or axis of symmetry brings the same pole rather than a mirror image pole in diametric structures (21).

While Lévi-Strauss treated these ancient, cross-cultural, interactive spatial systems as structures imbued with symbolic social meanings, his birds-eye view of cultures did not interrogate the individual’s lived experience in any given culture. These cross-cultural diametric and concentric spaces of relation have been extended to individual phenomenology in numerous domains, including education systems (49) and for social and emotional education (50). The current focus is on these shifting spaces as processes and structures of experience in AN, as diametric spatial systems shaping experiences of self, body, and other, to the exclusion of contrasting concentric spatial systems.

The interpretative spatial methodology in this article builds on a synthesis of a range of research traditions. In cognitive psychology and linguistics, conceptual metaphors and image schemas are uncovered in terms of background and foreground spatial understandings framing and shaping language, consistent with specific structures (51, 52). Another interpretative paradigm interrogating spatial background assumptions is Ricoeur’s hermeneutics (2, 37), where he is treated as offering a spatial turn for hermeneutics (38), including through spatially imbued fundamental terms such as distantiation and belonging (23, 30). Again, spatial structural dimensions here are argued at the level of consistency with spatial patterns and at the level of framing assumptions. Beyond cognitive psychology, linguistics, and hermeneutics, a further layer for this spatial questioning emerges from the structuralist anthropology of Lévi-Strauss. Here, Lévi-Strauss goes further to focus on contrasting spatial alternatives, such as concentric and diametric spaces, as spatial systems of meaning that are both explicitly manifested as physical structures and at a background level of interpretation in terms of social structures and myths (4, 47).

The current spatial interpretative methodology seeks to amplify the approaches of these conceptual traditions, through an interdisciplinary framework focusing on dynamic spatial frames (53) and malleable background system conditions of space (21, 49). In doing so, it seeks not only to argue for consistency between language, concept, and specific spatial structures but also to offer a tighter methodological scrutiny as direct contrasts in the spatial frames for language and concepts, between framing assumptions as diametric and concentric space, plus monistic space. Diametric space is explored as a candidate generative and maintaining condition for the restrictive experiential space underpinning AN phenomenology, as both a spatial process and structure shaping experience. This invites a clinical focus here on activating generative concentric spaces of assumed connection and relative openness in experience to restructure and replace diametric spaces.

## 4 Methodology

### 4.1 Selection criteria for reviewed phenomenological studies of AN

This desk-based research is a conceptual review of the literature, a theoretical paper. For illustration of the framework, studies were selected based on the criterion of salience in terms of diagnostic criteria for AN and central psychological themes in the AN literature. The selection criteria for the studies in this article were drawn from phenomenological work pertaining firstly to key DSM-5 criteria for anorexia nervosa, namely,

B. Intense fear of gaining weight or becoming fat or persistent behavior that interferes with weight gain.
C. Disturbed by one’s body weight or shape, self-worth influenced by body weight or shape, or persistent lack of recognition of seriousness of low body weight.

This includes the restricting anorexic subtype that “describes presentations in which weight loss is accomplished primarily through dieting, fasting, and/or excessive exercise” (54).

Allied with this diagnostic focus on drive for thinness and body dissatisfaction, the studies examined in this conceptual review give a phenomenological focus to central themes in the AN psychological literature regarding control, identity, and perfectionism. These studies comply with ethics requirements regarding informed consent and anonymity. The current focus was not on social media impact on AN or on comorbidities of AN or specific traumatic etiological factors for AN. The studies are illustrative, building on themes highlighted in a recent phenomenological metasynthesis of AN as a “state of being” [ (1), p. 5] and are not intended to be an exhaustive account of phenomenological studies and qualitative research themes for AN.

### 4.2 A phenomenology through space: levels of description

Key contrasts between diametric and concentric spatial systems offer a framework so that space is not only a unit of analysis but also a methodology for interrogating meaning in a range of empirical domains (21). Different levels of description pertaining to space are relevant in phenomenological accounts of AN, namely, space is being interrogated regarding a) direct spatial concepts, b) spatial imagery, and c) background frames for understanding, as taken for granted spatial assumptions in thought, emotion, and behavior. This third level of description here is with regard to diametric and concentrically framed background assumptions in the understandings of individuals experiencing AN. This level of spatial frames (53) or horizons of understanding has also been examined as the social imaginaries of individuals as conditions of experience (55). These dimensions are examined across the axis of contrasts between diametric and concentric spatial systems, building on the contrasts identified initially by Lévi-Strauss based on a range of cross-cultural anthropological observations of social structures and myths. The domain of relevance for Lévi-Strauss’ contrasts between diametric and concentric structured spaces is being expanded to the area of phenomenology. It is a phenomenology *through* space rather than *of* spatial perception.

### 4.3 Entailments of relative differences between diametric and concentric spaces

This proposed phenomenology through space builds from the key identified contrasts between diametric and concentric spatial systems in mutual interaction. These contrasts are regarding axes of assumed separation/connection, relative closure/openness, and mirror image inverted symmetry/symmetry, respectively (**Table 1**).

**Table 1:** Entailments of relative differences between diametric and concentric spaces.

Diametric spatial system hypothetical construct	Concentric spatial system hypothetical construct
Operationalization 1: assumed separation, splitting, exclusion	Operationalization 1: distinction on the basis of assumed connection
Operationalization 2: relative closure and non-interaction with background	Operationalization 2: relative openness and interaction with background
Operationalization 3: mirror image inverted symmetry	Operationalization 3: symmetry as unity

Elsewhere, a third candidate spatial mode has been identified that is outside the scope of the current study, namely, monistic fusion (56). This is experiential immersion in a sea of infinite stimuli, as obliteration of identity, a phenomenological dimension explored by Laing for schizoid experience (57). This monistic spatial mode of being is one of total openness, no boundaries, as both infinite space and empty space; it is an extreme within diametric spatial opposition where one pole totally dominates over the other opposing pole (56).

### 4.4 Addressing potential interpretative biases in the research methodology

Potential interpretative biases in this spatial framework include the inferential leap from hypothetical construct (e.g., diametric space) to its operationalization in a specific context as assumed separation or relative closure. This operationalization may be consistent with other spatial models and not only diametric space. It is this jump from observation to infinite possible hypothetical constructs that is highlighted as a key limit to traditional empirical methods (58). The necessary leap from observation, including qualitative phenomenological textual accounts, to the respective hypothetical constructs of diametric and concentric space is to be acknowledged as a limitation of all empirical research methods in the social sciences (58).

An example of the limited scope of this empirical and interpretative reasoning process can be taken from the cognitive science area of deduction. Johnson-Laird and Byrne attempt to show that people tend to reason deductively with mental models rather than formal logic rules. This is, strictly speaking, an argument at the relativistic level. This relativistic level is that x (mental models) is a better explanation than y (formal logic rules), rather than simply x is. They state that, “no amount of data, of course, can pick out one theory against all comers. Infinitely many theories are compatible with any finite set of observations. But, our problem is simpler: it is to decide amongst three possibilities” [ (59), p. 194], namely, formal rules, content-specific rules, and mental models for deduction. In cognitive science, Johnson-Laird and Byrne seek to resolve this problem by selecting between three purportedly mutually exclusive alternatives, in seeking grounds for preference of one hypothetical construct over another. Likewise, the proposed interpretative spatial methodology is one of *relative* preference for diametric spatial operationalizations over contrasting concentric spatial ones, as well as the third alternative monistic spatial reductions as fusion, infinite openness and empty space assumptions (56).

It is to be recognized that the empirical claim for assumed connection and concentric spatial relation, and assumed separation and diametric spatial relation, is at a relativistic level that one operationalization is more an expression of one spatial structure than the other one. It is their relative, relational differences that are being focused upon as referential inferences regarding these spatial structures. A strength of such referential inferences is that the spatial structures are visible and provide more concrete grounds for such publicly justifiable inferences than in relation to purely abstract hypothetical constructs. An argument for the “presence” of such spatial projections in empirical observations or other texts is strengthened if more than one of the relational entailments can be consistently uncovered in the texts. This brings empirical coherence across the entailments.

The spatial interpretative argument for a referential relation between concentric and diametric structures and the respective entailments of assumed connection and separation is a stronger one than simply that of consistency and concreteness. While other spatial structures or abstract concepts may also be linked with assumptions of connection and separation, cross-culturally observed concentric and diametric spatial structures are arguably more basic and elemental spatial structures than other ones, which might also provide such consistency (60). While other spatial structures may also be cross-culturally observed, a survey of anthropological research nevertheless concludes that “the simplest and at the same time most common type of symbolic classification … is the dual one” [ (61), p. 251]. Furthermore, concentric and diametric spatial structures offer a scope for a dynamic interplay between these spaces, in a way which may not be available to other static spatial models that are not so firmly conceptualized as being part of a unified whole.

Other spatial models would need to preclude the possibility that they are merely more complex versions of this basic opposition between concentric and diametric spaces. While parsimony of explanation is far from being a conclusive ground for preference of specific hypothetical constructs over others, it is this combination of being a more parsimonious explanation, with being a more dynamic, interactive and also basic structure of spatial relations, that provides other grounds for preference of a referential relation between concentric and diametric spaces and assumed connection and separation, respectively. Two different phenomenological levels are to be kept distinct to further avoid potential interpretative biases (60). The first level is the open-ended listening process to the lived experiential world of the individual, with the second, spatial phenomenological interpretative level taking place subsequently, through a different analytical source, to avoid a selection bias of the interviewer seeking responses from interviewees with AN to foreground spatial concerns with connection/separation, openness/closure, etc. The primary data, from which the secondary spatial analysis is based, are from independent phenomenological research sources.

It is also to be acknowledged that a potential bias and limit to a number of phenomenological studies of AN is the tendency to quote isolated passages from interviews that may be taken out of context for the meaning attributed to an individual, interviews that may lack thick description. A selection and construction takes place in the questioning process and foregrounding of textual extracts in qualitative phenomenological research, as well as in the narratives extracted from interview quotes of AN respondents. Nevertheless, it is well recognized that all empirical observation involves, to some degree, a process of theory-ladenness and construction, not only in the observational event recognition aspect but also its wider integration into a paradigmatic horizon of meaning recognition (62).

## 5 Results

### 5.1 Diametric space as opposition and splitting in AN: the anorexic voice and the gaze of the other

Notable phenomenological themes of the anorexic voice and the gaze of the other pertain to central aspects of AN regarding body dissatisfaction, identity, and drive for thinness. A key feature documented in phenomenology of AN is an “anorexic voice” that is in open conflict, in diametric opposition to the individual’s voice. In the phenomenological accounts of Williams and Reid (12):

participants experienced a split between their self and their disorder, which was experienced as a ‘battle’ between two minds or two separate voices. This experience was described by participants in varying terms; ‘anorexic voice’, ‘anorexic mind’, ‘Ana’, ‘anorexic thoughts’, ‘anorexic mode’, ‘negative tape in my head’ but all encompassed the same meaning of a critical, illogical and irrational entity that battled against and controlled their rational, true self. [ (12), p. 807].

This diametric oppositional space brings a split into antithetical forces in experience. In the words of Elizabeth, “I have a constant battle in my head between my voice and the voice of anorexia” and Jon, “it’s like there are 2 people in my head: the part of me that knows what needs to be done and the part of me that is trying to lead me astray. Ana is the part that is leading me astray and dominates me” [ (12), p. 807].

Participants viewed the anorexic thoughts as external (or alien) to the thoughts of their self. Their disorder was perceived as a separate entity that “took over” and “consumed” the person’s life and identity and made it difficult to escape [ (12), p. 807]. Diametric space is a process and structure of splitting of the self into a self in opposition to an alien other within. A different study of AN gave a similar finding where the body-as-object is not simply experienced as some inert object but has its own “voice” [ (11), p. 48].

These warring selves give expression to diametric oppositional space, as expressed in the following accounts, highlighted by Fuchs (10) from other works on AN, “It was as if I had to punish my body. I hated and detested it” [ (63), p. 278], “My body is not me” [ (64), Trans. Thomas Fuchs (10), p. 71]. Me and the not-me body are situated in a diametric oppositional spatial relation of mutual exclusion as assumed separation of side-by-sideness.

Accounts of not-being-at-home in one’s body, of a disconnection from the body, is a prevalent phenomenological feature in AN. This is again a hallmark of a diametric oppositional space and a loss of a capacity for connective concentric spatial relations of dwelling alongside. This is a loss of assumed connection to self, to the body experienced as the self. This may be a lack of capacity for the assumed connection of concentric space. It may also include a diametric oppositional relation to other people, as part of a withdrawal from social relations.

A further phenomenological aspect here is the individual with AN’s identity as perceiving their own body through the gaze of the other (31, 35), described by Hornbacher as “the habit of women with eating disorders perceiving themselves through others’ eyes, as if there were some Great Observer looking over their shoulder” [(65), p. 12]. Other phenomenological descriptions of AN individuals on this theme include statements such as “I feel my weight through the gaze of others,” “Others can make you feel beautiful and thin, or ugly and fat,” and “The gaze of other person gives me a sensation of being” [ (31), p. 147]. The other rests on the diametric oppositional spatial structure of assumed separation between self and other, as a precondition for the sense of reification of the body in this gaze.

### 5.2 Diametric space as a process of closure in the anorexic need for control: fear of freedom, avoidance, and the constriction of experience

Control and overexercise are well-established themes in AN and pertain to diametric spatial phenomenological processes of closure. A retreat into a mode of closure is a hallmark of phenomenology of AN. In the words of FG-Day P2, “I close off … sorry, that’s what I do … I close off and get into my little world … and I close off from the world.” [ (66), p. 19]. This is a diametric sealed space from background stimuli, of closed, non-interactive, hard borders, as a “thick partition” [ (67), p. 211], from the outside world, “Your intention was to become superhuman, skin thick as steel, unflinching in the face of adversity.” [ (65), p. 68]. Elsewhere, accounts of AN document a numbing “self-anestheticization” as a protective “shell” and “screen” [ (10), p. 113] against existence, while the account of Beth again highlights a stimuli reduction function of numbing “[F]or the most part, it STOPS me feeling. It numbs out pain, fear, anger, rejection.” [ (12), p. 804].

This attempt at cessation of stimuli through the rigid closed boundaries of diametric space is described by Rebecca as a process of freezing, “I am very good at freezing things out. (.) I could not bear to deal with it. I got extremely sad with it all. I did not take a position on it. Therefore, I simply froze it all out … I somehow tried to raise myself above it … and take it out on other areas such as exercising and not eating. That helps” [ (68), p. 53]. Frozen numbed walls of experience prevent fresh stimuli from entering the chamber of the experiential system; the closed domain of the diametric spatial system is exerted upon experience and propagates itself as a dynamic process of closure and non-interaction.

The certainty of closure as a spatial-existential mode of orientation is a search for rigid control:

FG-Day P3: Because part of my mentality is I have to get things fixed … I’ve got a notebook and I write down the things I have to do and I check it, score things off.
FG-Day P1: It’s like you are grabbing for something that sticks. If you’re worried or uncertain about something, you’re just grabbing for something that sticks…
FG-Day P1: Lists. That’s what I do and trying to do things that I’m familiar with. I need to get back into my routine, I have to go for a coffee with my mom because she can usually makes me feel … well, and it’s something familiar’. [ (66), p. 20].

Making lists is one such response to project a diametric space of closure to foster routines to forestall uncertainty.

A recent review of phenomenology of AN regarding overexercising accentuates this theme of an experiential retreat into a closed system, tantamount to a diametric spatial system, “the anorexic patient exercises a quasi-military regime over her body; she closes it off from exchange with the environment and suppresses its libidinal needs. She constructs her body as a self-contained, closed system” [ (10), p. 113] with sharp contours that resist openness and softness of fluid interaction.

Overexercise is described in phenomenological accounts as a way to close down emotions, to gain control over a fixed sealed world. This gives direct expression to diametric space as closure, “Hedda: In a way, it has been a bit like regulating feelings. If I am annoyed at someone or something, instead of somehow confronting them, or if I am sorry, or someone has said something … So in a way, it disappears as soon as I get out and start running” [ (68), p. 52].

These responses from AN inpatient focus groups point to a similar theme of suffocating closure and walls, implicating also a sense of breathing constriction:

‘FG-In P2: Build up inside, sort of where do you go with it, you just stick with it somehow, and you do, you do get through it. But it’s like someone suffocating you.
FG-In P1: Like the walls are caving in, like everything is tumbling down.
FG-In P2: Yeah, as if you are suffocated from the inside out.’ [(66), p. 15].

These accounts resonate with the view that “the act of self-starvation itself can be understood as an objectifying act in the sense that it diminishes the threatening demands of the body” [ (11), p. 51]. It reduces stimuli that threaten, stimuli from the body, as part of its spatial mode of closure, “an individual with AN does not experience her body simply as an overly large, inert, heavy object, but as a noisy, disruptive subject that threatens her own agency” [(11), p. 42]. An excess of stimuli, of “noise” from the body, needs to be closed down, to be sealed off. This is being proposed here as a diametric process of spatialization to minimize interaction with background stimuli, including those of the body. It is an avoidance mechanism spatially imbued. In the words of Agnes:

It is just that I think that, or I have come to believe that if I do not exercise, then everything will collapse … Then I do not quite know how to live, really. What should I do if I do not follow the routines I have taught myself? Therefore, it becomes a way to avoid thinking, or a period where I do not think, and I just do it. [ (68), p. 53].

Diametric spatial splitting and closure offer a mediating spatial process for avoidance. Routines fixated upon preserve the structure of a closed system of experience, a radical spatial filtering of fresh stimuli, whether stimuli emanating from the body, emotions, or thoughts. Routine may be a defense mechanism against the fluidity of more dynamic, porous spatial modes of experience, such as in concentric spaces of accelerated interaction with background stimuli. This AN strategy is one of an exaltation of diametric space as closure and cessation of the flow of more permeable concentric spatial boundaries for experience. It epitomizes loss of a capacity for a different spatial system of experience, of concentric spatial flow, in the retreat into the fixities of the hardened walled boundaries of diametric space.

The prevalent theme of control of uncertainty through a radical response of closure also emerges in this AN account, “FG-Day P3: To me the world is out of control and messy and horrific and that’s why, I wasn’t always aware of this, but that’s why I’ve lived more than half of my life in institutions because it reduces dramatically the amount of uncertainty that I’m faced with and chaos” [ (66), pp. 16–17]. Focus on weight offers a certainty for closure of self-definition, “When I have doubts about who I am, I go to my old friend: the Scale”, “I can’t tell exactly what my body is, I’m what the scale says about my body!” [ (31), p. 149]. While control is widely recognized as a key motivation for AN, it is the spatial concomitants of such control, as a diametric process of spatialization to shut down from interaction with stimuli, that requires further recognition. Diametric spatial modes of experience and loss of being-in-the-world as side-by-sideness become the reactive operative mode of coping through this compression into a closure of experiential space. Restriction of food in AN may be part of a wider process of spatial constriction as the operation of a diametric spatial system of experience.

This diametric spatial closure from stimuli is expressed by a 32-year-old Australian woman with severe disordered eating for more than half her life, through the imagery of a rabbit hole:

When in a rabbit hole, you become largely blind (or nonchalant) to those and the world around you. You are too far down to see them much or properly. And it is all too comfortable, familiar and interesting for you to care otherwise … Go away people, and just leave me to it. [ (8), p. 1].

Some accounts describe a fear of taking up space, of opening up a spatial domain, “it is easier to go for a run and get it out through that. I then, in a way, have been spared from having taken up any space” [ (68), p. 52]. Again, this is resonant with a fear of engaging in concentric space’s more permeable open boundaries. Concentric spaces of experience allow a more expansive range of stimuli to gain entry to experience, whereas an anorexic response of withdrawal and closure is the antithesis of this, as a quest for diametric spatial modes of being. The accentuation of diametric space devours the mediating assumed connection and more dynamic open interaction with background of concentric space.

### 5.3 Diametric space as mirror image inversions of love/hate ambivalence and perfectionist success/failure in AN

Ambivalence is a recurring theme in the phenomenology of AN that is relevant to key AN diagnostic concerns with body dissatisfaction, while identity concerns with perfectionism pertain to diagnostic issues of drive for thinness. This ambivalence occurs in relation to oneself and also to the eating disorder itself. Ambivalence is a diametric spatially structured mirror image inverted symmetry, exemplified also as love/hate and confident/insecure:

“It is, at the most basic level, a bundle of deadly contradictions; a desire for power that strips you of all power. A gesture of strength that divests you of all strength.” [ (65), p. 6].

Jon states, “Ana saved my life when I couldn’t cope, but is now killing me … and I know it, but can’t stop it.” [ (12), p. 805].

“…the constant change of emotion, up down, up down, up down, the constant battle in your head, back and forth, back and forth … the confusion that you often feel about yourself … how you feel about recovery, how you … you know you’re constantly changing minds I guess….” [ (69), p. 27].

Other accounts of perfectionism associated with AN point to the central lived experiential importance of the success/failure mirror image inversion. The perfectionist dimension commonly associated with AN is construed as “fear of failure” [ (12), p. 805], building on the words of Beth, “I have always been a high achiever and perfectionist. Never got to grips with the fact that I couldn’t always be the best. [ … ]. Guess I thought if I couldn’t be the best musician/top of my class/prettiest/whatever, I could maybe be the thinnest” [ (12), p. 805]. This diametric spatial mirror image inversion of success/failure is a strong phenomenological preoccupation in the literature on AN, highlighted as a diametric spatial system feature elsewhere as a logic of performativity in education systems (21). A variant of this diametric mirror image spatial inversion is above/below hierarchy manifested in this quote, “Why I starve myself? Because of all the people in my life who die of jealousy when they see the way I look” [ (31), p. 147]. Stanghellini and Mancini emphasize “two main constructs pertaining [to] disorders of identity” in AN [ (31), p. 147], namely, severe clinical perfectionism and core low self-esteem (70). Diametric spatial mirror image inversion offers here a candidate maintaining condition for these constructs.

This account of Rebecca gives strong expression to the dominion of a diametric spatial mirror image inversion in her preoccupation with winner/loser and success/failure identities:

With identity then, it is a bit that I have based my self-esteem on obtaining good performances in sport. That is why when at first, if I had a bad competition I was completely crushed, because I felt that ‘what else do I have’. (.) It was the training, kind of, I felt good then and if I performed well in competitions. I do not feel I have anything else. I feel that I need something concrete, that it will be black and white how good you are in sports and things like that. In a way, I do not consider being a good friend to be … I had to obtain all my confidence from performing well. And when I performed well, life was all fantastic. When I performed badly, then it was incredibly awful. Then the world might as well have come to an end. [ (68), p. 56].

This need to shift away from diametric reactive spaces of splitting, closure, and mirror image inversions as ambivalence invites a question as to how to promote concentric spaces in experience, how to shift toward concentric spatial habits of experience, as a more fluid capacity for experience. Concentric spaces of assumed connection such as trust, compassion, and some aspects of non-competitive play are part of a challenge to the Cartesian spatial habit of self-consciousness resting on diametric spaces of assumed separation that make the self an object for itself.

Other questions arise here as to how embodied spaces, spaces experienced in the body as frozen and rigid can be fluidated toward more open concentric spatial flows. These spatial concerns are preverbal and precognitive though impacting on verbal and cognitive systems, as well as emotions. The preverbal dimension of these spatial experiences is aptly expressed by the AN inpatient “FG-In P2: Yeah, as if you are suffocated from the inside out. You can’t explain to somebody what the feeling is like; you can’t explain it to yourself let alone to somebody else” [ (66), pp. 15–16].

This is echoed in different AN accounts:

‘It [running] really helps to get rid of something one cannot say in words …’ [ (68), p. 52].
‘this project of control need not be conceived of in terms of a cognitive, cold project but rather as a habitual, pre-reflective set of embodied practices that the individual with AN adopts in response to the intolerable tension she experiences between her body-as-subject and body-as-object’ [ (11), p. 52].

This prereflective dimension to belonging, pertinent to AN as a capacity for experience, is one accentuated by Ricoeur, though not explicitly as a concentric spatial belonging. Concentric and diametric spaces are a candidate system of tensions between opposing spatial directional movements that are prior to language and shape experience (**Table 2**).

**Table 2:** Manifestations of diametric space in the phenomenology of AN.

Diametric space operationalization 1: assumed separation, splitting, exclusion	Relevant to central AN DSM-5 diagnostic concerns with body dissatisfaction, drive for thinness, overexercise, and central psychological issues in AN of control, identity, and perfectionism
Framing assumption of separation, splitting, exclusion	Battle between two separate voices—process (identity, drive for thinness) Preverbal exclusion as getting rid of something—process (body dissatisfaction, drive for thinness) Intolerable tension between body as subject and body as object—process (body dissatisfaction, drive for thinness) Body is not me—structure (body dissatisfaction, identity) Gaze of the other—process (body dissatisfaction, identity)
Diametric space operationalization 2: system of relative closure and non-interaction with background	
Space as a direct concept of closure	I close off from the world—process (control, identity) Grabbing for something that sticks, get things fixed—process (control) Diminish threatening noise from bodily stimuli—process (control, body dissatisfaction) Closes it off from exchange, self-contained closed system—structure (control, identity)
Imagery of closure	Skin thick as steel, thick closed boundaries—structure (control, identity) Protective shell and screen—structure (control, identity) A rabbit hole—structure (control, identity) Freezing things out—process (control) Like something suffocating you—process (body dissatisfaction) Like the walls are caving in—process (body dissatisfaction, control)
Framing assumption of closure	Numbs out stimuli of pain—process (control, body dissatisfaction) Get back into my routine—process (control, overexercise) Collapse of everything if not following self-taught exercise routines—process (control, overexercise) Live in institutions to dramatically reduce uncertainty—process (control, identity) Exercise to be spared from taking up space—process (control, overexercise) Preverbal feeling of being suffocated—process (body dissatisfaction)
Diametric space operationalization 3: mirror image inverted symmetry	
Framing assumption of mirror image inversion	Desire for power that strips of power—process (control, identity, drive for thinness) Saves life but now killing me and cannot stop it—process (control, identity, drive for thinness) Not best at music, academics or most beautiful so be best at being thin—process (perfectionism, drive for thinness) Need something concrete in black and white—structure (control, identity) Make others jealous as being above them—structure (identity, perfectionism, drive for thinness)

## 6 Discussion

### 6.1 Pervasive features of diametric space in the phenomenology of AN

Building on the growing research on the phenomenology of AN in recent decades, it is remarkable how consistently the phenomenology of AN clusters around structural features of diametric space. This proposed spatial framework integrates an apparently diverse, diffuse range of experiences associated with AN in phenomenological research. Pervasive features of AN experience and motivation, well established in the international research literature, such as need for control, perfectionism, an anorexic “voice,” and self-hate around the body, are all aspects that cluster around a specific spatial system and process, namely, diametric space. Diametric spatial closure from background stimuli is a spatial concomitant of control. Perfectionism is associated with binaries of success/failure as diametric spatial mirror image inversions, and the anorexic voice as an assumed separation from the rest of the self, as a diametric spatial opposition between self and body. Diametric spatial mirror image inversions serve as operative processes in love/hate ambivalence. Features of closure from stimuli in freezing, numbing, and restricted breathing in the phenomenology of AN further exhibit diametric spatial systemic dimensions of experience. It can be concluded that key aspects of AN experience are motivated by the exigency of a recurring spatial system movement of diametric space, across multiple systems of experience pertaining to overexercise, control, identity, and perfectionism, including realms associated with key DSM-5 diagnostic dimensions for AN regarding drive for thinness and body dissatisfaction.

These structural features of the lived experience of a range of individuals with AN, based on examples from AN phenomenological research across a range of studies, interpreted in spatial terms, point to a recurring coping mechanism, central to anorexic experience, as one that seeks diametric spaces as a state of being. This also points to the exclusion of the directly contrasting concentric spatial mode of experience, where, for example, diametric spatial splitting excludes concentric spatial connection on a particular dimension, and diametric spatial closure opposes concentric spatial processes of relative opening.

Understanding diverse aspects of the phenomenology of AN as this key recurring diametric spatial system response invites a further focus on AN recovery in terms of shift from these diametric spatial system habits of defensive experience toward contrasting concentric spaces of experience as framing projections for thoughts, feelings, behavior, and breathing, as part of concern with capacity for interaction between diametric and concentric spaces mediating experience of self and world. A recurrent clinical concern implicated through the focus on background generative and maintaining spatial processes shaping the phenomenology of anorexic restriction is that it is not only important to adopt a foreground focus in AN on food restriction and body image but additionally on the wider holistic experiential background constrictions. This suggests a related focus on promoting the wider background capacity for concentric spatial processes in experience, as momentum toward developing and restructuring connection and openness for self, body, and eating.

### 6.2 Key issue of diametric spatial closure as maintaining condition for control

In the range of phenomenological accounts reviewed, the diametric spatial dimension of closure is a particularly prevalent phenomenon, both in amount and across the three levels of description as direct spatial concepts, as imagery, and as framing assumptions. Thus, in a proposed phenomenological shift toward concentric spaces of experience to help AN recovery, challenge to the diametric spatial process of closure may be predicted to offer the most pertinent starting point to develop a capacity for multilayered, staged, relative experiential openings, such as a phased capacity for novelty and uncertainty, as well as for attunement to experience of emotions beyond numbing and expanded breathing beyond a compression of space as suffocating. Osler highlights how hunger from the visceral body can replace unwanted feelings, “through self-starvation, one can saturate the body with a controlled and deliberate experience of hunger which can take the place of other unwanted feelings” [ (39), p. 69]. This key stimuli reduction process of closure also for excluding feelings is phenomenologically resonant with a diametric spatial process, combining both closure and splitting as a dynamic process of assumed separation. However, to the extent that Osler emphasizes a saturation function of hunger, as an increase in stimuli even to counter other stimuli, this is in tension with the stimuli reduction process of the closure of diametric space, one attested also in the phenomenological accounts of numbing and freezing. The restriction of emotional function of diametric spatial operations is less a saturation of feeling than its filtering for reduction and elimination. It is, as described elsewhere by Osler in AN phenomenology, a controlling of the noise (11), a sealed disconnection, and not a replacement of one noise through acceleration of another.

The fundamental phenomenological desire for control in AN rests on a potentially malleable spatial condition of diametric space framing experience, as both a spatial process and structure; diametric space offers a candidate maintaining condition for control as a state of being. The spatial restriction in the closure of diametric space provides a maintaining condition for the restriction of food in the body in AN. It appears that the spatial axis of closure versus opening in the diametric–concentric space interaction is a key phenomenological starting point for engaging in recovery from AN.

### 6.3 Shift from diametric space to concentric space for AN recovery

While it is recognized that “AN is marked by a clash between different dimensions of bodily experience” [ (11), p. 54], a further step needs to be taken, to construe AN in terms of a dynamic clash between mutually interactive spatial systems of experience, namely, diametric and concentric spaces of relation. This invites construal of these diametric spaces as being reactive and compensatory, as a loss of capacity for concentric structured spatial experiences. The examples of diametric space in framing assumptions, direct conceptual understandings, and imagery in AN phenomenology invite the question as to whether these diametric spatial system constellations in experience are causal dimensions to the development of AN, or maintaining conditions to ensure its persistence, or simply epiphenomena, mere incidental by-products. This requires further examination in terms of focus on system shifts away from diametric spatial habits of experience and toward concentric spaces of being. It also requires further analysis as to whether these diametric spatial system responses are factors to be associated with trauma generally, with specific manifestations taking place for AN. More focus is also needed on whether the proposed key system response of diametric space is a global concept for experience in AN or is better construed as a range of specific diametric spatial subsystems impacting in specific ways on emotion, visceral body, thought, and behavior.

This spatial phenomenological paradigm proposes that it is not simply a matter of challenging entrenched diametric spatial modes of experience but also of offering transitional spaces, namely, concentric spatial processes to mediate assumed connection and relative openness as a preverbal capacity underpinning and shaping the individual’s perceptual, somatic, emotional, breathing, cognitive, and social domains. While recognizing the ambivalence of patients to engage in treatment (71), a goal in the process of recovery in AN is to fluidate the rigid diametric spatial fixations constellated through these various aspects of the individual’s system of lived experience. This treats AN recovery as not only a matter of weight gain but additionally a wider capacity to generate concentric spaces of holistic life experiences and preintentional existential feeling (36), restructuring from diametric spatial oppositions, splitting, closure, and inversions as ambivalence.

Stanghellini and Mancini’s recognition that in AN “what seems to be impaired is the coenaesthetic apprehension of their own body as a more primitive and basic form of self-awareness” [ (31), p. 146] is strongly resonant with the need for a state of being in concentric space of assumed connection with self and body, prior to diametric spatial juxtaposition and distantiation as a prerequisite for self-conscious reflexivity. Both approaches share a concern with capacity for modes of experience, pertaining to unmediated prereflexive and self-conscious experiential modes. Moreover, they both envisage a displacement from prereflexive experience in AN. The proposed spatial phenomenological approach in this article examines the background generative and maintaining conditions for the modes of experience identified by Stanghellini and Mancini (31).

A recovery focus on generating multilayered concentric spaces of experience invites engagement with activities outside success/failure diametric mirror image performativity, less self-conscious spaces where the self is not subject to the distantiation process of being an object for oneself. Concentric spaces of being are needed for experience where the individual is not evaluating self in relation to other, whether this identity of difference is construed in diametric spatial hierarchical terms as better or worse than others; a prereflexive space of experience is needed, of preintentional existential feeling, as a capacity prior to the reference point of judgment of and by others. This shift from a mode of assumed separation in judgment is allied with a shift from the mode of the visual sought by Stanghellini (32), while also seeking to reconstruct from diametric spatial projections constraining the ambit of the visual for wider more fluid soothing visual stimuli. These spaces of belonging to connect with one’s body and possibly meditation to help expand constrictions and suffocations of breathing involve spaces and activities of experiential flow and play as assumed connection to self, body, and others. Treating diametric spaces as a defensive compensatory system, as loss of concentric spatial relation, there is a need to move beyond diametric spaces of self-evaluation (success/failure) and not simply to seek other diametric domains of self-evaluation beyond weight and shape. Concentric spaces of connective experience are needed outside of a self-conscious mode of self-evaluation itself, outside competitive, performative modes of experience and activity. Concentric space of relative openness fosters capacity for uncertainty, a relevant goal even initially in other spheres of experience, unrelated to eating as such. Building on strengths, this holistic capacity for a plurality of concentric spaces of mediation may enable an experiential shift influencing movement from problematic diametric spaces of experience. This includes challenge to meaning making in rigid diametric oppositions and to diametric spaces of assumed separation that make the self an object for oneself.

A clinical focus on cognitions to challenge rigid diametric spatial oppositions invites a focus on opening to complexity beyond diametric mirror image good/bad thinking. This is not only for thin equals good and weight gain equals bad type of diametric spatial frames but also for a wider capacity to construe other cognitions and emotions with complexity and lack of certainty, to sensitively engage with diverse contextual stimuli. A clinical starting point here is to help overcome sweeping attributions of good/bad for other domains of the individual’s experience and world. Rigid diametric mirror image inverted spatial cognitions in AN pertain not only to good/bad but also success/failure. Again, there is a need to open up, fluidate, and soften these rigid diametric spatial closures of constriction to recognize diversity of successes and to challenge rigid internalizations of failure identity.

A related challenge to diametric spatial closure embedded in activity (as well as cognitive) routines that is of potential clinical relevance here includes a concern to open from routines generally and to face the anxiety in doing so. This capacity to engage with novelty invites challenge not only to routines around eating or exercise but also other specific individual routines as spatial modes of closure restricting experience. There is a need to confront and overcome the anxiety of change to minor, less significant routines in order to build up strength to change for major routines pertaining to food restriction, including overexercise.

Novel environments and activities offer a relative opening from the restriction of stimuli in diametric space. This challenges not only wider AN desire for control, including not only control of food, self, and body, but the diametric spatial closure filtering novel stimuli, as part of the process of insulation of self in control. Clinically relevant pathways for progress here in AN phenomenology to challenge diametric closure of space structuring experience are developing capacity for engagement with less predictable environments, with novel unexpected stimuli, as part of a capacity for experiential stretching into concentric space, for loosening the Procrustean bed of diametric space. These experiential openings may not be focused initially on food or the body but on wider environments, activities, and experiences. This can include a perceptual level of expansion through immersion in natural environments, in watching movements where fixity as such is fluidated, such as watching the waves of the sea or rivers.

A somatic clinical focus here again focuses on this capacity to engage more with stimuli. Leder’s visceral body concerns (33) pointing to recovery pathways for AN through dance, movement therapy, massage, yoga, or tai chi (34) are of direct relevance here, as are opportunities for swimming in a supported environment and art therapy for an expanded attunement to perceptual stimuli to challenge the constricted diametric perceptual space of the gaze of the other. Going further to a concern with breathing constriction, meditation offers another avenue for this experiential opening and connection. Fostering concentric spatial assumed connection to body and self can build on Leder’s concern for modes of healing through “lived experiences of bodily care, relaxation, harmonious attunement and pleasure” [ (34), p. 60]. This can further embrace a non-threatening capacity for connection and care with animals and plants, plus a capacity for flow of happiness and love without guilt or shame (35) in the space of the body, through non-instrumental, non-goal-oriented activity, in order to just be, for a prereflexive concentric space of being.

This accelerated clinical focus on diametric spatial system blockages constraining experience and concrete pathways to open from these through concentric spaces offers a framework for dialogue with the individual experiencing AN and for plans to strategize for personal growth.

### 6.4 Uncovering the diametric and concentric spatial background frames for the gaze of the other in AN phenomenology

The Sartrean gaze of the other has been given emphasis in a range of AN phenomenological accounts on identity (31, 35). Yet this is a synthesis of at least six components: the other, othering as a process of separation, the visual dimension of the gaze, the reifying objectification in this gaze, a reification not only of self but also of body, and the conception of the other as a non-caring, non-compassionate other. A spatial phenomenological account treats the dimension of objectification of the Sartrean gaze of the other felt by the person with AN in relation to their own body and self as a compound of different spatial components and processes. It highlights the different spatial aspects for the proposed integration of cognitive behavioral therapy and phenomenological models of eating disorders that focus on “breaking the pathological interconnection between impaired coenaesthesia, imprisonment in an allocentric perspective on one’s own body and overreliance on the gaze of the others, as the only way through which defining one’s own identity” [ (72), p. 2520]. This proposed core feature of AN (16) phenomenology requires integration with background spatial assumptions.

To do so, firstly, it is to be noted that De Beavoir’s initial conception of the other, in terms of gender, explicitly drew on Levi-Strauss’ work (73). De Beauvoir’s characterization of woman as “other” explicitly relied on Lévi-Strauss’ “profound work” [ (73), p. 17] on diametric oppositions, though not emphasizing spatial aspects (56). De Beauvoir’s *The Second Sex* explicitly states that “the category of the other is as primordial as consciousness itself” [ (73), p. 16] and thereby as preceding the duality of the sexes. The duality of diametric spatial opposition between self and other is the precondition for the very conception of the other, and this goes beyond gender-based terms, as de Beauvoir explicitly recognizes (56).

The other rests on the diametric spatial structure of us versus them, of assumed separation between self and other. De Beauvoir treats the other as a category, “otherness is a fundamental category of human thought” [ (73), p. 17]. Yet it is not simply a category of the other but a diametric spatial relation of distantiation. Going further, the other is additionally an active process of othering within the self, a diametric spatial process of splitting within, of separation of self in the role of being an observer of oneself. In diametric spatial experience, the body is reified as a juxtaposed object counterstanding to oneself. Here, diametric space is both a maintaining condition for the other and a generative process of being an enabling condition as a spatial movement for othering. Stanghellini emphasizes the reliance on the visual modality in this gaze of the other within oneself (32). The distantiation needed for sight of objects is supported by diametric spatial oppositions juxtaposed side-by-side, as a precondition for objectification. Thus, restructuring from the visual may need to also engage with a restructuring from diametric space habits of experience, as part of a developing a fluid mobility in the individual’s experiential system.

The diametric space underpinning the gaze of the other is, if not always a hostile one, nevertheless, a distantiation from self as assumed separation of a typically impersonal, non-caring other, rather than a concrete loving other in a concentric relational space of assumed connection. Clinical implications of this spatial phenomenological reading of the compound spatial features of the gaze of the other impacting on experience of the body in AN include a focus on promoting prereflexive unmediated concentric spaces of being and existential feeling, of being in the body, as well as reconfiguring the diametric spatial modes bringing reliance on the visual. Yet this is not an avoidance of all visual realms for experience, for a diametric spatial closure off from visual stimuli, but rather a shift from evaluative visual realms bringing judgment of diametric mirror image success/failure. Moreover, it includes an emotional experiential focus on compassionate concrete others for internalization in the sense of self and body, to shift from the diametric spatial background framing reification and distantiation of the abstract other in the Sartrean gaze.

These features of splitting as opposition, closure, and mirror image inversions build on the diametric spatial systems identified initially cross-culturally by Lévi-Strauss (4, 47) for physical and social structures, as well as myths and expanded subsequently into individual phenomenology and educational systems (21, 49, 50). This article is the first to apply these spaces specifically to the phenomenology of AN. The current framework prioritizes the prereflective background capacity for relation itself, as a spatial phenomenon; here, different spatial processes and structures shape contrasting experiences of self and body, as well as being a mediating space between subjectivity and objectivity. This treats the capacity for being in relation, in preverbal, precognitive spatial terms, as malleable, shifting modes of diametric and concentric spatial systems in dynamic interplay.

The proposed spatial phenomenological framework provides a more precise analysis than the loose metaphors of transparency versus opaqueness as subjective versus objective experience in AN (74), as it gives a spatial background rationale for permeability of more open boundaries in concentric space and the harder, non-interactive system space with background in more closed, and hence opaque, diametric relation. Moreover, it takes experience out of the rigid subject–object schema for a more dynamic interactive background of generative spaces shaping experience, as a dynamic system.

A pertinent phenomenological issue for AN is the entrenched imbalance toward diametric systems of experience in various manifestations and lack of capacity for concentric spaces of experience in many ways. Displacement within experiential space, from concentric to diametric space, is proposed as a prior system paving the way for the bodily tensions in AN; change to spatial conditions for bodily experience can help toward embodiment. The interplay between a diametric and concentric spatial phenomenology is not being proposed as a feature of spatial experience unique to AN. It has been interrogated elsewhere as general features of human experience (21) and also regarding specific spatial imbalances in schizoid phenomenology (60). It is the particular kinds of imbalances in this spatial interplay—an extreme propensity toward diametric spaces and suppression of concentric spaces—that bear the hallmarks of a phenomenology pertaining to AN.

### 6.5 Issues for future spatial phenomenological research and limitations of the framework

The phenomenological accounts explored here are not intended to be exhaustive and would be strengthened through more specific research on phenomenology regarding differences in age, time in history, gender, and cultural contexts. Spatial phenomenological differences in chronicity of AN may also be pertinent, while recognizing also that responsibility for the chronicity of AN may be due to inefficacy of treatments than the disorder itself (75). Limitations of the current article include that it has not sought to directly engage with psychodynamic aspects in their relation to phenomenology, or with issues of specific comorbidities, trauma, family system dynamics, life events, and social media or directly with phenomenology of sexuality for AN.

A developmental focus for AN (76) may also be needed to distinguish attachment histories where an individual’s insecure attachment (77) meant that the capacity for concentric spaces of connection was not established in early childhood (60). This would predict a greater difficulty in sustaining concentric spatial modes of experience than when a loss of this experience occurs later in life. A temporal dimension to examine changing phenomenological trajectories in AN recovery invites prediction of shifts from diametric to concentric spaces of experience across these three axes: assumed separation/connection, relative closure/openness, and mirror image inverted symmetry/symmetry.

A potential limitation to the multidimensional system lens employed for the spatial phenomenology of AN is that it does not offer a guide to which system lens, if any, to prioritize regarding change from diametric spaces. This priority may be co-constructed as part of a dialogue between the individual with AN and the clinician. The precise interplay between these spaces as maintaining conditions or additionally generative spatial processes contributing to the establishment of AN needs further examination. Its role as framing spatial preconditions does not explain individual differences. More research is also needed on any relative priority for clinical impact regarding focus on change to the open-closure spatial axis, over, for example, the assumed connection–separation axis in concentric and diametric spatial interaction in individual experience for AN. Again, this may be a matter of dialogue with the individual. More research is needed on how to bring these shifts from diametric to concentric space in AN over time and individual resistances to doing so. Nevertheless, this spatial phenomenological framework goes further than simply a typical phenomenological focus on meaning construction to also engage with causal trajectory issues for change in AN, through treating space as a malleable, sustaining system condition in experience, relevant to central diagnostic and psychological understandings of AN.

As well as being examined in future research, this spatial movement away from diametric spaces of being and toward concentric spaces of being offers a pathway to inform clinical practice.

To conclude, this spatial phenomenological paradigm proposes that the phenomenology of AN is fundamentally a spatial crisis of loss of connective modes of spatial openness embedded through concentric spaces of experience, and loss of belonging, in the shift toward the radical oppositional distantiation, objectifying juxtaposition and closure of diametric spaces of experience projected into understandings, emotions, the visceral body, and behaviors. AN as a diametric spatial side-by-sideness needs a fundamental experiential shift in capacity to engage through more open, connective concentric spaces.

## Data Availability

The original contributions presented in the study are included in the article/supplementary material. Further inquiries can be directed to the corresponding author.
